# Proposing Two Local Modeling Approaches for Discriminating PGI Sunite Lamb from Other Origins Using Stable Isotopes and Machine Learning

**DOI:** 10.3390/foods11060846

**Published:** 2022-03-16

**Authors:** Ruting Zhao, Xiaoxia Liu, Jishi Wang, Yanyun Wang, Ai-Liang Chen, Yan Zhao, Shuming Yang

**Affiliations:** 1 Institute of Quality Standard and Testing Technology for Agro-Products, Chinese Academy of Agricultural Sciences, Beijing 100081, China; zhaoruting1201@163.com (R.Z.); lxxiagood@163.com (X.L.); wjs2018@163.com (J.W.); 82101182143@caas.cn (Y.W.); ailiang.chen@gmail.com (A.-L.C.); zhaoyan01@caas.cn (Y.Z.); 2 Key Laboratory of Agro-Product Quality and Safety, Ministry of Agriculture, Beijing 100081, China

**Keywords:** local modeling, protected geographical indication, Sunite lamb, stable isotopes, machine learning

## Abstract

For the protection of Protected Geographical Indication (PGI) Sunite lamb, PGI Sunite lamb samples and lamb samples from two other banners in the Inner Mongolia autonomous region were distinguished by stable isotopes (δ^13^C, δ^15^N, δ^2^H, and δ^18^O) and two local modeling approaches. In terms of the main characteristics and predictive performance, local modeling was better than global modeling. The accuracies of five local models (LDA, RF, SVM, BPNN, and KNN) obtained by the Adaptive Kennard–Stone algorithm were 91.30%, 95.65%, 91.30%, 100%, and 91.30%, respectively. The accuracies of the five local models obtained by an approach of PCA–Full distance based on DD–SIMCA were 91.30%, 91.30%, 91.30%, 100%, and 95.65%, respectively. The accuracies of the five global models were 91.30%, 91.30%, 91.30%, 100%, and 91.30%, respectively. Stable isotope ratio analysis combined with local modeling can be used as an effective indicator for protecting PGI Sunite lamb.

## 1. Introduction

Sunite sheep were formed in the special ecological environment of Sunite grassland through long-term natural selection and artificial selection. They enjoy natural herbage and pure water in the ecological environment of natural grassland without pollution, and feed on more than 400 kinds of natural herbage, such as *Allium mongolicum regel*, *Allium polyrhizum turcz*, and *Stipa capillata*. It is this good ecological environment and primitive and extensive feeding mode that afford Sunite lamb with excellent quality and flavor [1,2]. Sunite lamb was awarded protected geographical indication (PGI) status in China in 2008. PGI Sunite lamb originates from the Sunite Right Banner and Sunite Left Banner, and it recognizes its high-quality reputation and characteristic flavors. Therefore, PGI labeling guarantees the origin and quality of food products, minimizing food safety risks, and ensures consumer confidence for the declaration of origin on this commodity [3].

In order to protect the PGI products, researchers have put forward a fingerprint tracing method, that is, using chemical parameters to build the fingerprint of geographical indication products [4] and comparing it with the fingerprint of the testing sample to determine whether the testing sample is the geographical indication product. At present, the chemical parameters used in the traceability of animal-origin food include stable isotopes [5], mineral elements [6], fatty acid content [7,8], amino acid content [9], and metabolites [10]. Stable isotopes are commonly used to characterize geographical origin information and to describe agricultural products’ origin information, where δ^2^H and δ^18^O can be used to distinguish altitude, δ^15^N can be used to determine the type of grazing vegetation, and δ^13^C can determine the type of animal feed [11]. In addition, δ^34^S is related to rainfall in the geological environment and traditional industrial emissions, so it indicates the geographical characteristics of animal food. Sr is obtained from the decay of ^87^Rb, and its stable isotope abundance is mainly affected by geological conditions and rock ages. Sr has good applications in plant-derived-food tracing [12], but it is limited in tracing the origin of animal food due to its low content in animal bodies [13].

Thus, the stable isotope ratios can be used to distinguish PGI Sunite lamb from different origins. Stable isotopes have been applied to determine the origin of different animal-origin foods, such as beef [14], lamb [15,16], milk and dairy products [17], and marine products [18]. In 2007, Camin et al. [19] measured δ^13^C, δ^15^N, δ^2^H, δ^18^O, and δ^34^S in crude lamb protein from 13 European regions, and achieved correct classification rate of original grouping and cross validation of 78.7% and 77.6%, respectively. This indicated the feasibility of using stable isotopes to distinguish the geographical origin of lambs. However, the information of the samples Camin et al. [19] tested was complex, such as samples collected in different years from the same region, and samples collected in the same year for different feeding methods from the same region, meaning that the sample set covered a wide range of variations, which led to the model’s lower predictive performance. On the other hand, the wide range of variations in the sample set may cover samples that will appear in the future; that is to say, it is conducive to improve the prediction ability of the model for unknown samples. Additionally, smaller sample difference coverage also leads to lower predictive performance. In the study by Sun et al. [5], the similarities of feed types, agricultural practice, and environment in two regions accounted for the overlapping of lamb samples from these two regions in the Inner Mongolia autonomous region. In subsequent studies, in order to improve the prediction ability of the geographical origin model, not only was the increase of chemical parameters considered, but also the coverage of sample differences.

In previous research on food traceability, the global modeling method was used to establish the discriminant model, that is, to create a model from all data sets that cover the whole space [20]. However, a good traceability model requires that the sample set should cover as wide a range as possible and avoid the appearance of samples with as similar chemical information as possible. In addition, the number of samples in the model should not be too large, so as to avoid the increase in interference information along with the increase in information, which will reduce the prediction performance of the model [21]. In fact, in the field of the near-infrared spectrum, scholars have focused on the coverage and representativeness of the sample set [22,23,24]. In a large sample set, there is a nonlinear relationship between response Y and all predictors X to varying degrees, and a sample set with a linear relationship can be obtained based on distance similarity. This is local modeling, where a set of local model data is created from all data sets according to certain rules, each covering a subspace [20]. Local modeling includes two rules; one is selecting the local model data set based on spatial similarity, and the other is selecting the most representative data subset based on the uniform design principle. Abhinav et al. [25] used the small spectral library obtained by a local modeling scheme based on spatial similarity to predict the soil property parameters of samples, which improved the prediction accuracy of soil properties compared with global modeling. This local modeling scheme referred to predicting the response of the samples by finding the most similar samples from existing databases. The similarity here was based on distance measures, such as the Euclidean distance, the covariance distance, the correlation distance, the surface difference spectrum, the information distance, optimized principal component Mahalanobis distance, and local linear embedding. Additionally, sampling representative samples can ensure that the chemical parameter characteristics and property range of the sample set can better cover the chemical parameter properties of unknown samples and improve the prediction ability of the unknown samples. In 2017, Palou et al. [26] proposed a strategy for calibration set selection of biodiesel/diesel samples based on principal component analysis (PCA) and the Kennard–Stones algorithm, and the results showed that, by using this methodology, the models could keep their robustness over time. In the future, local modeling should be more applied in the discrimination of the geographical origin of agricultural products.

In order to better discriminate PGI Sunite lamb from other origins using stable isotopes (δ^13^C, δ^15^N, δ^2^H, and δ^18^O) and machine learning, we proposed two local modeling approaches to optimize the sample set. It is worth mentioning that this is the first exploration of the protection of PGI Sunite lamb, and also a new application of local modeling in origin identification. The two local modeling approaches were (a) the Adaptive Kennard–Stone (AKS) algorithm and (b) an approach of PCA–Full Distance (FD) based on Data-Driven Soft Independent Modeling of Class Analogy (DD–SIMCA). The AKS algorithm was used to select the most representative data subset based on a uniform design principle, and the approach of PCA–FD based on DD–SIMCA was used to select the local model data set based on spatial similarity. It should be emphasized here that global modeling and local modeling in this study refer to the selection of data set coverage space, and the establishment of the discriminant model still depends on machine learning. The machine learning methods used in this work are linear discriminant analysis (LDA), random forests (RF), support vector machine (SVM), back-propagation neural network (BPNN), and k-nearest neighbor (KNN) classification. Based on the confusion matrix, we compared the predictive performance of the traceability models established by the five machine learning methods.

## 2. Proposed Two Local Modeling Approaches

### 2.1. Adaptive Kennard–Stone (AKS)

AKS is an adaptive sample selection method based on the Kennard–Stone algorithm, and its advantage is that it can determine the optimal sample set. The idea of AKS is to provide a uniform spatial design for the selection of the most representative samples from the known sample set. It ensures that the chemical parameters and property range of the sample set can better cover that of the unknown samples, and improves the prediction ability of the unknown samples. To our knowledge, there was only one report related to AKS application in the near-infrared spectrum [23]. At present, AKS has not been reported in the discrimination of the geographical origin of agricultural products, but Kennard–Stone (KS) has been reported [27].

**Figure 1:**
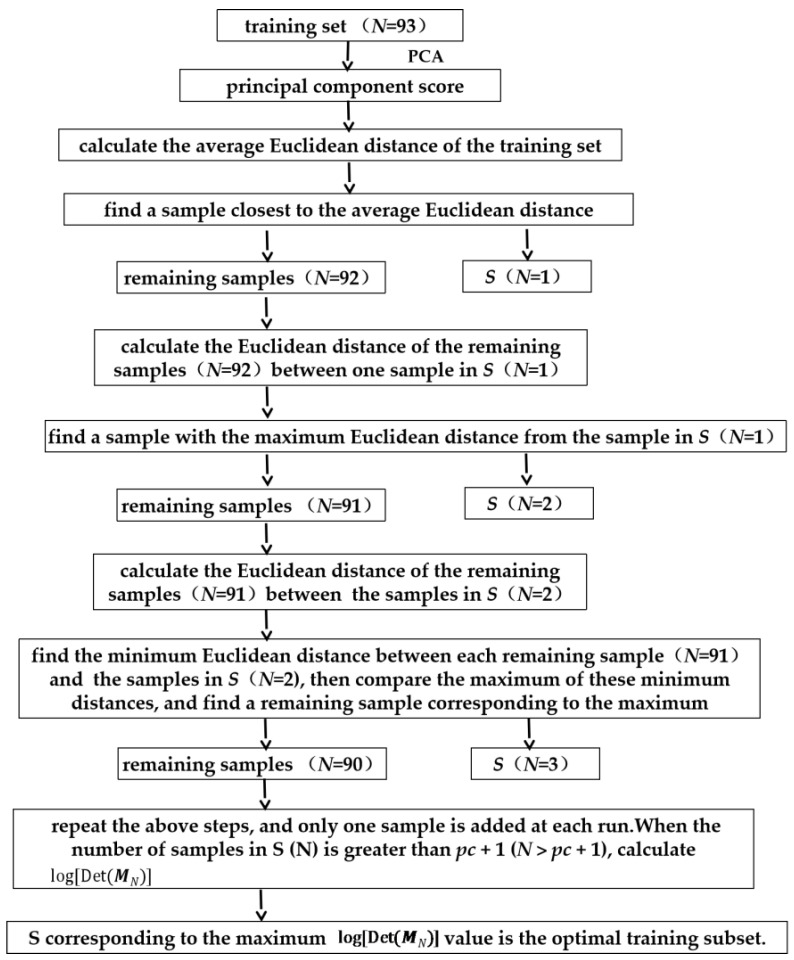
The steps of the AKS algorithm.

The D-optimal criterion [28] was used as the criterion to select the samples. The minimum variance in the model could be achieved by selecting the right number of samples included in ***S*** that maximize $\log\left[\mathrm{Det}\left(M_{N}\right)\right]$. The $\log\left[\mathrm{Det}\left(M_{N}\right)\right]$ can be represented as the information of the selected sample set. The one we chose was the subset with the most information per sample, which was given by $\log\left[\mathrm{Det}\left(M_{N}\right)\right]$, where ***M****_N_* = ***S***^T^***S***/*N*, Det is the determinant of the matrix, and ***S*** is the principal component score matrix of the selected sample set. The number of principal components was *pc*. Figure 1 shows the steps for obtaining the optimal sample set [23].

### 2.2. An Approach of PCA–Full Distance (FD) Based on Data-Driven Soft Independent Modeling of Class Analogy (DD–SIMCA)

The measurement of similarity used to be based on Euclidean distance, Mahalanobis distance, principal component analysis Euclidean distance (PCA–ED), and principal component analysis Mahalanobis distance (PCA–MD). Both distances can be calculated in the original variable space and in the principal component space. In the principal component space, the correlation between variables is eliminated, simplifying the data information and making it superior to the original variable space. The Euclidean distance is the straight line distance between two points, and it is affected by the data distribution, noise, and characteristic metrics. Unlike the Euclidean distance, the Mahalanobis distance introduces a covariance matrix, and implements coordinate rotation and data compression, which makes Mahalanobis distance not affected by data distribution and feature dimensions [29]. However, these distance threshold choices were hard, often selected several times, and then compared the predictive ability of the model of the selected multiple sample sets. It took time and effort to achieve this, but the best sample set might not be found. Moreover, the whole process could not be visualized, making it harder to understand. Based on the understanding of DD–SIMCA, we found that PCA–FD integrated the advantages of MD and ED, the operation process was simple, and the results were visible. Therefore, we proposed an approach of PCA–FD based on DD–SIMCA. As far as we know, an approach of PCA–FD based on DD–SIMCA to screen the samples has not been reported.

Each element of the data cloud can be presented as a sum of two vectors: a vector that lies in the subspace (a projection) and a vector transversal to the hyperplane (a residual). The lengths of these vectors are important indicators that characterize a sample position with respect to the subspace (model). These statistics are often referred to as the leverage and the residual variance. In DD–SIMCA, they are termed as the score distance (SD) and the orthogonal distance (OD) correspondingly, which are used to define the critical limits of the classification model [30]. SD is equal to the squared Mahalanobis distance from the model center to one sample within the score subspace, and OD is the squared Euclidean distance from one sample to the model subspace. FD is affected by parameters related to SD and OD (See Formula (1)), as shown below.

(1) $$FD=\frac{Nh\times SD}{SD0}+\frac{Nq\times OD}{OD0}$$

where *Nh* and *Nq* are the degrees of freedom (DOF) for SD and OD, and SD0 and OD0 are the means of SD and OD of all the samples [31], respectively. PCA–FD integrated the advantages of MD and ED and eliminated the effects of data distribution and feature dimensions. Moreover, the approach of PCA–FD based on DD–SIMCA simplified the operation and could be visualized. As shown in Figure S1, the abscissa and ordinate of the acceptance plot were the parameters associated with SD and OD, respectively, and the boundary lines of regulars and outliers are given. The red line and the green line were available on the figure, and the yellow line was added later. The red line is the boundary of the outliers, and samples above the bounds are outliers; the green line is the boundary of regulars, and samples below the bounds are regulars; the points in the middle area of the green line and the red line are extremes; the yellow line is FD. The FD on the same line is the same, and the larger the FD, the farther the yellow line is away from the base point. You can obtain all samples inside the strip centered on the testing sample (a black triangular), such as a custom bound of

*FD*_1_ < *FD* _one testing sample_ < *FD*_2_ (2)

where the border values, FD_1_ and FD_2_, have certain rules. Samples between the two yellow lines are the screened samples.

As a measure of similarity, FD was used to select samples similar to one testing sample from the original sample set to solve the nonlinear problem of large-sample data modeling. This approach greatly simplifies the threshold selection process, and part of the process is visualized.

## 3. Predictive Performance of the Model

The predictive performance of the model in our work based on the confusion matrix includes the sensitivity, specificity, accuracy, and kappa coefficient (these measures were calculated for each method based on the test data set). The confusion matrix summarizes the results of a classification method. For a binary classification, when we determine that class 1 is positive, the schematic table of the confusion matrix is shown in Table 1.

In this study, instead of negative and positive, the classes were “non-PGI lamb” and “PGI Sunite lamb”, respectively. For example, TN is the number of non-PGI lamb in the test data set correctly classified as non-PGI, and FN is the number of PGI Sunite lamb incorrectly classified as non-PGI. The sensitivity, specificity, accuracy, and kappa coefficient are defined as follows:

(3) $$sensitivity=\frac{TP}{TP+FN}$$

(4) $$specificity=\frac{TN}{TN+FP}$$

(5) $$accuracy=\frac{TP+TN}{TP+TN+FP+FN}$$

(6) $$kappa=\frac{P0-Pe}{1-Pe}$$

where P0 (P0 = accuracy) indicates the accuracy of the model, and Pe ($Pe=\frac{\left(TP+FN\right)\times \left(TP+FP\right)+\left(TN+FN\right)\times \left(TN+FP\right)}{(TP+TN+FP+FN{)}^{2}}$) is the expected proportion of lamb correctly classified by chance.

Sensitivity is the proportion of actual PGI Sunite lamb that is correctly classified as PGI Sunite lamb. Specificity is the proportion of actual non-PGI lamb that is correctly classified as non-PGI lamb. Accuracy is the ratio of true positive and true negative samples to the total number of testing samples, which reflects the overall accuracy. If the proportion of one class of samples is not dominant in all classes of samples, then its high error rate has little influence on the accuracy. In this case, the accuracy does not carry much meaning, and the Kappa coefficient can better reflect the discrimination effect. The Kappa coefficient can better reflect the consistency of actual classification and predict classification. The evaluation result of the Kappa coefficient is divided into three grades [32]: excellent (Kappa > 0.75), good (0.40 < Kappa ≤ 0.75), and poor (Kappa ≤ 0.40). As long as the accuracy of one class is low, the Kappa coefficient will decrease.

## 4. Materials and Methods

### 4.1. Materials

**Figure 2:**
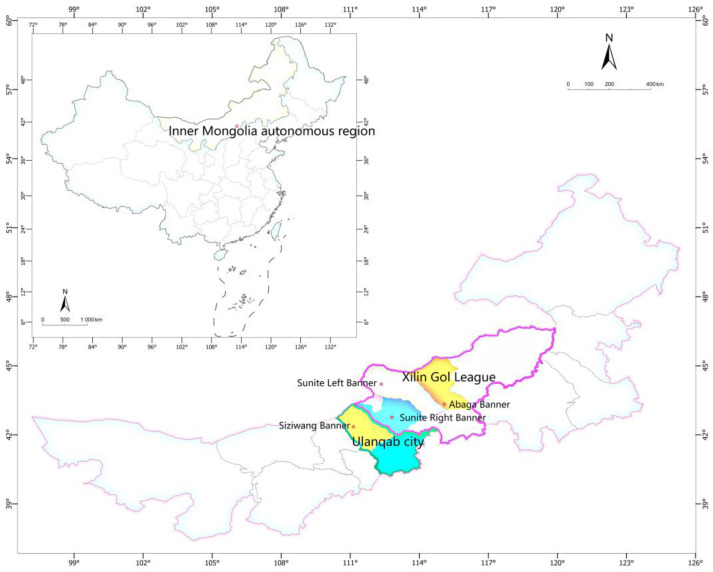
Regional location information for the lamb samples.

Lamb samples (*n* = 116) were collected from 4 banners in two cities of China’s Inner Mongolia autonomous region (Table S1), where the Sunite Right Banner and Sunite Left Banner are the specified regions of PGI Sunite lamb, located in Xilin Gol League; Abaga Banner also belongs to Xilin Gol League, east of Sunite Left Banner; Siziwang Banner belongs to Ulanqab City, west of Sunite Right Banner (Figure 2). The lamb samples from each banner came from the same abattoir and were collected from the right hind leg. The samples were from 5–8-month-old grazing sheep. The fresh mutton (50 g) was dried to a constant weight and then pulverized through a 100 mesh. The sample was mixed with a chloroform/methanol (2:1, *v*/*v*) solution at 1:5, vortexed for 10 min, and centrifuged at 5000 rpm for 5 min, and the supernatant was discarded [33]. Then, the previous degreasing step was repeated twice, the supernatant was discarded, and the solid was retained and lyophilized to obtain a defatted dry matter (DDM) for the determination of stable isotopes. These samples were stored at −20 °C for subsequent analysis.

### 4.2. Stable Isotope Analysis

For the stable isotope analysis of δ^13^C and δ^15^N, DDM and other international reference materials (USGS40, USGS43, and USGS62) were weighed into tin capsules (5 × 8 mm) and then introduced into an elemental analyzer (Flash 2000, Thermo, Waltham, MA, USA), converting the entire material into carbon dioxide and nitrogen gas analyzed by an isotope ratio mass spectrometer (Delta V Advantage of Thermo, Waltham, MA, USA). The calibration of δ^13^C and δ^15^N was analyzed with USGS40 (δ^13^C = −26.39‰, δ^15^N = −4.5‰ air N2), USGS43 (Indian Hair, δ^13^C = −21.28‰, δ^15^N = 8.44‰), and USGS62 (caffeine, δ^13^C = −14.79‰, δ^15^N = 20.17‰).

For the stable isotope ratio analysis of δ^2^H and δ^18^O, DDM and international reference materials (Caribou Hoof, Kudu Horn, and EMA P2) were weighed into silver capsules (4 × 6 mm) along with other international reference materials and introduced into the elemental analyzers (Flash 2000, Thermo, Waltham, MA, USA). The reactor packing was a glassy carbon reactor and silver wool. The elements hydrogen and oxygen in the samples were converted into H_2_ and CO at 1380 °C via pyrolysis with glass carbon. The gas was transferred to an isotope ratio mass spectrometer (Delta V Advantage, Thermo, Waltham, MA, USA). The calibration of δ2H and δ18O was analyzed with CBS (Caribou Hoof Standard, δ^2^H = −197.00‰, δ^18^O = 3.80‰), KHS (Kudu Horn Standard, δ^2^H = −54.10‰, δ^18^O = 20.3‰), and B2205 (EMA P2, δ^2^H = −87.80‰, δ^18^O = 26.90‰).

The results of the isotope analysis were expressed as δ (‰), and the formula was

(7) $$\delta \;\left(‰\right)=\frac{Rsample-Rstandard}{Rsample}\times 1000$$

where R sample and R standard are the isotope ratios of the sample and the international reference material, respectively. The references of δ^13^C, δ^15^N, δ^2^H, and δ^18^O were Vienna–Pee Dee Belemnite (V–PDB), Air, Standard Mean Ocean Water (SMOW), and SMOW, respectively.

### 4.3. Statistical Analysis

All of the samples (*N* = 116) were divided into a training set and a testing set (4:1). Due to the uneven sample size in the four regions, stratified random sampling was adopted to avoid contingency, and samples in each region were divided into 4:1. The training set samples (*N* = 93) were used for modeling, and the testing set samples (*N* = 23) were used to evaluate the prediction ability of the model. The training set data were imported into R Studio and the training set subset (*N* < 93) was obtained by the AKS algorithm. After that, the training set subset used five machine learning methods (LDA, RF, SVM, BPNN, and KNN) to establish the geographical origin discriminant model, and finally used the confusion matrix of testing set samples to evaluate the predictive performance of the model.

One sample (called *Pi*, *i* = 1, 2, 3, …, 23) in the testing set (*N* = 23) and the training set samples (*N* = 93) was imported into DD–SIMCA in Microsoft Excel (SIMCA template-xlsb) to obtain the FD of all samples (*N* = 93), and the training subset was appropriately selected centering on the FD of *Pi.* Then, import the training set subset into R Studio and use the 5 machine learning methods to build a one-time local model for *Pi*. Repeat the above operation 23 times, and obtain 5 confusion matrices. The evaluation method of the model was consistent with the evaluation method of the training set subset obtained by the AKS algorithm.

All of the training set samples (*N* = 93) were imported into R Studio, and 5 machine learning methods were used to establish the geographical origin discriminant model. The evaluation method of the model was consistent with the evaluation method of the training subset obtained by the AKS algorithm.

In order to compare the changes before and after screening the training set samples (*N* = 93), we analyzed the main characteristics of the global lamb isotope libraries and local lamb isotope libraries obtained by the AKS algorithm. SPSS was used to conduct an independent-samples T-test to analyze the significance between the two groups (PGI Sunite lamb and non-PGI lamb), produce box diagrams to intuitively see the significance, conduct exploratory analysis (mean value, standard deviation, and histogram), and produce the 3-dimensional scatter plot to observe the spatial distribution of the sample set. Furthermore, to know the difference of lamb between different regions, we performed a descriptive analysis of all data (*N* = 116) (Table S2). Additionally, we drew a 3D–score plot of the global lamb isotope library and local lamb isotope library according to geographical origin (Figure S2).

The statistical software packages R, SPSS 25.0 (SPSS Inc., Chicago, IL, USA), and a chemometric tool employed in Excel were used. AKS was written by our laboratory using R language.

## 5. Results and Discussions

### 5.1. Training Subset Obtained by Two Local Modeling Approaches

In this work, the training subset was obtained by two local modeling approaches: (a) AKS and (b) the approach of PCA–FD based on DD–SIMCA. Based on the AKS algorithm, a line chart (Figure S3) was drawn using the $\log\left[\mathrm{Det}\left(M_{N}\right)\right]$ value as the ordinate and number of samples as the abscissa. As shown in Figure S3, when the number of samples was 40, the maximum $\log\left[\mathrm{Det}\left(M_{N}\right)\right]$ value appeared, and the corresponding S subset was the best training set subset. Chen et al. [23] used AKS to screen the near-infrared spectrum library of plant alkali, and 49 samples were selected from 85 samples for constructing the PLS model. The sample size was also half of the original data. However, Chen et al. [23] continued to sample the near-infrared spectrum library of the aqueous solution and selected 37 samples out of 38 samples to construct the PLS model [23]. This shows that the capacity of the training subset was not related to the capacity of the training set, but was only related to the information contained in the training subset, namely the $\log\left[\mathrm{Det}\left(M_{N}\right)\right]$ value. When the maximum $\log\left[\mathrm{Det}\left(M_{N}\right)\right]$ value is not reached, the $\log\left[\mathrm{Det}\left(M_{N}\right)\right]$ value increases with the increase in the number of samples. When the maximum $\log\left[\mathrm{Det}\left(M_{N}\right)\right]$ value is reached, the $\log\left[\mathrm{Det}\left(M_{N}\right)\right]$ value decreases as the sample size increases.

According to the approach of PCA–FD based on DD–SIMCA, 23 targeted training subsets were obtained with a sample size between 20 and 47. When screening data, we found that, when the FD of *Pi* (Figure S1a) deviated from the central position of FD of all data (*N* = 93), the discriminant effect of the model established by the data set with a small sample size (20 ≤ *N* < 35) was better, and that when the FD of *Pi* (Figure S1b) close to the central position of FD of all data, the discriminant effect of the model established by the data set with a medium sample size (35 ≤ *N* < 50) was better. This may be related to the principle of screening. This method selected samples within the linear range of *Pi* for modeling based on similarity [22]. The linear range was probably related to the position of the FD of *Pi* in the FD of all of the data. In the future, this finding will continue to be verified in order to summarize the screening rules.

To sum up, during sample screening, it is necessary to follow the screening principles and consider the data characteristics to select data with an appropriate sample size. In this work, with two local modeling methods, half or less of the original sample size could be used to obtain the same model effect as the original data.

### 5.2. Main Characteristics of the Lamb Isotope Libraries

**Figure 3:**
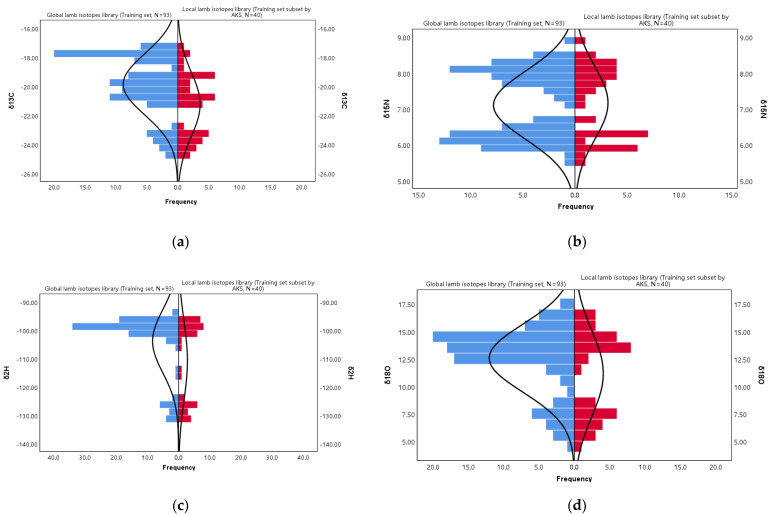
Histograms of the δ^13^C (**a**), δ^15^N (**b**), δ^2^H (**c**), and δ^18^O (**d**) values of the global lamb isotope library and local lamb isotope library.

Taking the training set subset (*N* = 40) obtained by AKS and the training set (*N* = 93) as an example, the main characteristics of the local and global lamb isotope libraries were compared. Table 2 lists the mean, the standard deviation, and the ranges spanned by the samples and Figure 3 shows the corresponding distribution histograms. For the mean of δ^13^C, δ^2^H, and δ^18^O, the local lamb isotope library was smaller than the global lamb isotope library. For the standard deviation of δ^13^C, δ^15^N, δ^2^H, and δ^18^O, the local lamb isotope library was larger than the global lamb isotope library. The mean reflected the overall average and the degree of data concentration, while the standard deviation reflected the degree of data dispersion. This meant that the local lamb isotope library was more centralized and more dispersed, and was the ideal training set. The histogram (Figure 3) also supported this conclusion. Through the histogram, we could see the data distribution of the local and global lamb isotope libraries more intuitively. The distribution of the global lamb isotope libraries was not uniform, and some data were abrupt in the histogram, which had a great influence on the main characteristics of the whole data set. The local lamb isotope libraries weakened the influence of prominent data and better reflected the main characteristics of the overall data.

**Figure 4:**
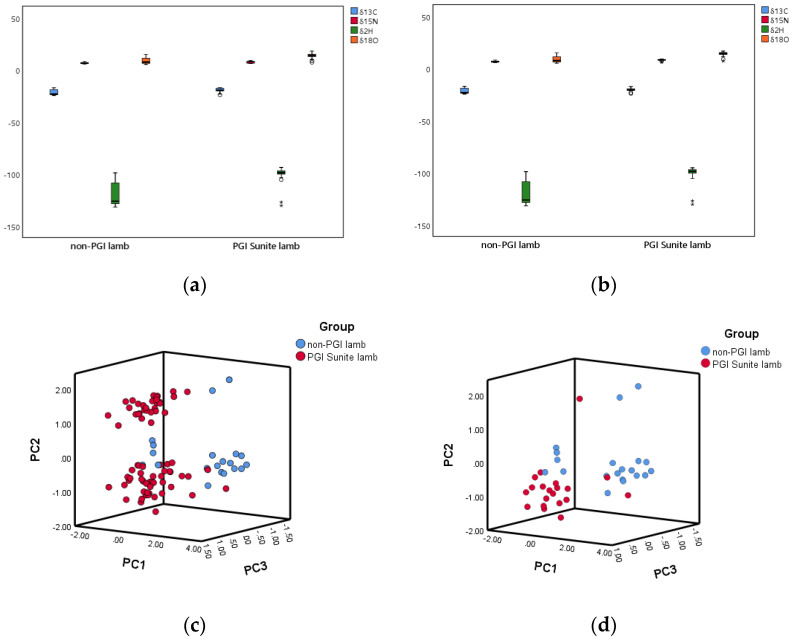
Boxplots of the δ^13^C, δ^15^N, δ^2^H, and δ^18^O values of (**a**) global lamb isotope library and (**b**) local lamb isotope library according to lamb groups; 3D–score plot of (**c**) global lamb isotope library and (**d**) local lamb isotope library according to lamb groups.

In this work, we paid more attention to the influence of screening on the two categories, rather than the overall data. The significance of isotopes has an impact on the accuracy of geographical origin discrimination and traceability feasibility [34], so we compared the significance of the δ^13^C, δ^15^N, δ^2^H, and δ^18^O values between PGI Sunite lamb and non-PGI lamb in the local and global lamb isotope libraries. The result of the T-test showed that there were both significant differences (*p* < 0.05) in the δ^13^C, δ^15^N, δ^2^H, and δ^18^O values between PGI Sunite lamb and non-PGI lamb before and after sample screening. This meant that PGI Sunite lamb and non-PGI lamb always had a characteristic stable isotope ratio profile. Figure 4a,b show the corresponding boxplots, and consistent conclusions could be drawn. In the local and global lamb isotope libraries, PGI Sunite lamb samples exhibited the highest δ^13^C, δ^15^N, δ^2^H, and δ^18^O values. The regional disparity of the δ^13^C and δ^15^N of lamb samples was a consequence of the feeding systems [35]. The δ^13^C value in animal products was based on C_3_ and C_4_ plants in the animal diet. One study showed that the δ^13^C value of C_3_ plants ranged from −20‰ to −35‰, and that the δ^13^C value of C_4_ plants ranged between −9‰ and −17‰ [36]. In this work, the lamb samples were grazing sheep. We could predict that the proportion of C_3_ and C_4_ plants fed to PGI Sunite lamb was higher than that fed to non-PGI lamb. In another aspect, the value of δ^15^N reflects the nitrogen cycle in soil. Compared with other C_3_ plants, leguminous plants can directly utilize atmospheric nitrogen, resulting in a lower δ^15^N value [37]. Generally, leguminous plants, such as alfalfa hay, are abundant at high altitudes, which could be the cause of lower δ^15^N value of lambs (non-PGI lamb) from high-altitude regions, such Siziwang Banner (Table S2). The values of δ^2^H and δ^18^O reflect the geographical information of lamb, such as altitude. In the atmospheric circulation process, the higher the altitude, the lower the enrichment degree of ^2^H_2_O, and the δ^2^H and δ^18^O values in the high-altitude region are lower than those in the low-altitude region [38]. The best examples are Sunite Left Banner and Siziwang Banner in Table S2. The altitude of Sunite Left Banner is higher than that of the four sons king flag (Table 2), and the δ^2^H and δ^18^O in the lamb of Sunite Left Banner are significantly lower than those in the lamb of Siziwang Banner. In addition, the values of δ^13^C, δ^15^N, δ^2^H, and δ^18^O are affected by objective factors, such as rainfall, temperature, and geology.

We carried out descriptive analysis and independent-samples T-tests on the local and global lamb isotope libraries above, and concluded that sample screening can optimize the sample data. Now, we can more intuitively determine the data spatial distribution and whether the data are representative through the 3D–score plot. Additionally, we can also determine the contribution of the δ^13^C, δ^15^N, δ^2^H, and δ^18^O values to PC1 and PC2 (Figure S4). In the 3D–score plot of the global lamb isotope library (Figure 4c), PGI Sunite lamb and non-PGI lamb samples overlapped and were difficult to distinguish. From Figure S2a, the lamb samples from Abaga Banner adjacent to the PGI area had a serious overlap with PGI Sunite lamb. However, in the 3D–score plot of the local lamb isotope library (Figure 4d), PGI Sunite lamb samples were entirely separated from the non-PGI lamb samples. Additionally, the samples from the four regions were completely separated (Figure S2b). After sample screening, the spatial distribution was uniform and the samples were representative. These results provide strong evidence that the local lamb isotope libraries were superior to the global lamb isotope libraries.

### 5.3. Predictive Performance

According to the above data feature analysis, the local lamb isotope libraries were better than the global lamb isotope libraries. The significance analysis and 3D–score plot showed that it was feasible to use isotopes to discriminate PGI Sunite lamb from non-PGI lamb. To further compare the local and global lamb isotope libraries, machine learning was used for modeling, and some indicators of predictive performance were used to evaluate the effect of the model. For the same library, five machine learning methods were used to ensure model stability.

The origin classification results of applying the five models to the testing set lambs are shown in Table 3, together with the evaluation of prediction performance. The evaluation of each binary discriminant model was built from the confusion matrix, the records of which correctly and incorrectly recognized samples from different geographical origins. True positives were samples of PGI Sunite lamb correctly predicted, false negatives were samples of PGI Sunite lamb incorrectly predicted to be from non-PGI lamb, true negatives were samples of non-PGI lamb correctly predicted to be from non-PGI lamb, and false positives were samples of non-PGI lamb incorrectly predicted to be from PGI Sunite lamb. The evaluation of the whole model was calculated as a two-class overall classification.

As shown in Table 3, in the global lamb isotope libraries, the five models established by machine learning achieved a good discrimination effect. Among them, the confusion matrix of models established by LDA, RF, SVM, and KNN was all one false negative and one false positive; that is to say, a sample of PGI Sunite lamb was incorrectly predicted to be from non-PGI lamb, and a sample of non-PGI lamb was incorrectly predicted to be from PGI Sunite lamb. The sensitivity and specificity of the above models were 94.44% and 80.00%, respectively. Additionally, the accuracy of the model was 91.30%, a satisfactory result for the overall classification. The Kappa coefficient was 0.7444 (0.40 < Kappa ≤ 0.75), a good consistency of actual classification and predict classification. On the other hand, the confusion matrix of the BPNN model was true positives and true negatives, and all the classes were correctly discriminated. This indicates that the five origin models established based on global lamb isotope libraries were stable, and the BPNN model had the best predictive performance.

In Table 3, using local lamb isotope libraries screened by AKS, the five models established by machine learning had a better discriminating effect, in which the order of predictive performance was LDA, SVM, and KNN < RF < BPNN. It was the same as the predictive performance of four models (LDA, SVM, BPNN, and KNN) based on the local and global lamb isotope libraries. Compared to global modeling, locally modeled RF models are superior to globally modeled RF models. In the RF model, only one PGI Sunite lamb was incorrectly predicted to be from non-PGI lamb, and all of the non-PGI lamb samples were identified as non-PGI lamb (specificity = 100.00%). The accuracy of the RF model was 95.65%, a very satisfactory overall classification result. Additionally, the Kappa coefficient was 0.8808 (Kappa > 0.75), indicating excellent consistency of actual classification and prediction classification. In other words, the local lamb isotope libraries obtained by AKS were better than the global lamb isotope libraries. The KS algorithm has also been applied to Protected Designation of Origin (PDO) cheeses recently, and good results were obtained. In 2021, Coppa et al. found that mid-infrared spectroscopy (MIR) enables the authentication of the cow feeding restrictions included in the specification of two PDO cheeses (Cantal and Laguiole). The classification result of the testing sample showed that the accuracy, sensitivity, and specificity of Cantal PDO cheeses were 90.3%, 91.1%, and 89.2% respectively; and the predictive performances of the model for Laguiole PDO cheeses were 99.5%, 100%, and 99.4%, which all outperformed the AKS modeling effect in this paper [27]. However, it must be said that Coppa et al. used the KS algorithm to select training sets and testing sets. We consider it inappropriate to select the testing sets, because it changes the true distribution of the sample, which may be the reason for the over-good classification results.

There was a better discrimination effect of five models using local lamb isotope libraries screened by the approach of PCA–FD based on DD–SIMCA (Table 3), in which the order of predictive performance was LDA, RF, and SVM < KNN < BPNN. It was the same as the predictive performance of four models (LDA, RF, SVM, and BPNN) based on local and global lamb isotope libraries. Compared to global modeling, locally modeled KNN models were superior to globally modeled RF models. In the KNN model, only one PGI Sunite lamb was incorrectly predicted to be from non-PGI lamb, and all of the non-PGI lamb samples were identified as non-PGI lamb (specificity = 100.00%). The accuracy of the KNN model was 95.65%, a very satisfactory result overall classification. Additionally, the Kappa coefficient was 0.8808 (Kappa > 0.75), indicating excellent consistency of actual classification and prediction classification. In other words, the local lamb isotope libraries obtained by the approach of PCA–FD based on DD–SIMCA were better than the global lamb isotope libraries.

The differences in the isotope profiles of the lamb’s geographical origins allowed satisfactory discrimination between them, but were not sufficiently wide and systematic to be validated by adding an external set sample to the classification model. As shown in Figure 2, the lamb samples collected were PGI Sunite lambs and non-PGI lambs in their adjacent origins; that is, lambs at municipal geographical distance from the PGI Sunite lamb and lambs at banner geographical distance from the PGI Sunite lamb. This is because the geographical information difference of lambs at the provincial level and above is large and easy to distinguish [5]. Thus, this study pays more attention to the identification of lambs at municipal/banner/county geographical distances. After that, lambs from other provinces and countries would be added to enrich the sample library so that the sample library could cover as large sample differences as possible, such as geographical origin, feeding type, breed, age, and gender differences, and the external samples were verified.

To sum up, the local lamb isotope libraries obtained by AKS and the approach of PCA–FD based on DD–SIMCA were better than the global lamb isotope libraries.

## 6. Conclusions

In this work, stable isotope ratio (δ^13^C, δ^15^N, δ^2^H, and δ^18^O values) analysis combined with local modeling was used to discriminate PGI Sunite lamb from other origins, and the accuracy rate reached 100%, which could be used as an effective indicator system for protecting PGI Sunite lamb. A good traceability model requires that the sample set should cover as wide a range as possible and avoid the appearance of samples with basically the same chemical information as much as possible. Therefore, local modeling is very necessary for the traceability of agricultural products, but it has not been reported. In this paper, two local modeling approaches were first proposed for the protection of PGI Sunite lamb, and the identification effect of models was better than that of global modeling, which could be used for the optimization of the training set and the traceability of agricultural products. We found that the sample set with less than or equal to half of the original sample size in this study could achieve a better predictive effect. However, the ratio of the screened sample size to the original sample size will not always be 1:2, and the screened sample size is related to the information contained. The information (geographical origin, feeding system, age, and gender) of the lamb samples collected in this paper was similar. This may account for the small changes in the data characteristics before and after screening. In the future, while increasing the sample size, we will try our best to make the sample set cover a wide range of differences, such as geographical origin, feeding system, breed, age, and gender. At that time, there will be a more obvious nonlinear relationship between the classification response and the isotope ratio, and the application of a local modeling method is more necessary.

## Figures and Tables

**Table 1:** The schematic table of the confusion matrix.

Confusion Matrix		Predicted Class	
		Class 1	Class 2
Actual class	Class 1	True positive (TP)	False negative (FN)
	Class 2	False positive (FP)	True negative (TN)

**Table 2:** (**a**) Descriptive statistics of the isotope attributes of samples in the lamb isotope libraries. (**b**) δ^13^C, δ^15^N, δ^2^H, and δ^18^O values of the local and global lamb isotopes libraries from two groups.

(**a**)				
**Parameter**	**Mean**	**Standard Deviation**	**Minimum**	**Maximum**
Global lamb isotope library (Training set, *n* = 93)				
δ^13^C	−19.87	2.10	−24.69	−17.16
δ^15^N	7.10	0.95	5.58	8.88
δ^2^H	−103.90	11.14	−131.87	−93.77
δ^18^O	12.55	3.08	4.92	17.98
Local lamb isotope library (Training subset by AKS, *n* = 40)				
δ^13^C	−21.24	2.21	−24.69	−17.26
δ^15^N	7.16	1.01	5.58	8.88
δ^2^H	−110.85	14.11	−131.87	−95.44
δ^18^O	11.15	3.90	4.92	16.56
(**b**)				
**Parameter**	**δ^13^C**	**δ^15^N**	**δ^2^H**	**δ^18^O**
Global lamb isotope library (Training set, *n* = 93)				
PGI Sunite lamb	−19.31 ± 1.52 ^a^	7.28 ± 0.94 ^a^	−99.48 ± 5.44 ^a^	13.65 ± 1.90 ^a^
non-PGI lamb	−21.92 ± 2.65 ^b^	6.45 ± 0.70 ^b^	−120.02 ± 11.84 ^b^	8.53 ± 3.28 ^b^
Local lamb isotope library (Training subset by AKS, *n* = 40)				
PGI Sunite lamb	−20.57 ± 1.44 ^a^	7.88 ± 0.73 ^a^	−101.68 ± 9.58 ^a^	13.78 ± 2.43 ^a^
non-PGI lamb	−21.92 ± 2.65 ^b^	6.45 ± 0.70 ^b^	−120.02 ± 11.84 ^b^	8.53 ± 3.28 ^b^

Note: The values are given as mean ± SD; the small letters represent significant differences (*p* < 0.05); the sample sizes of Sunite Right Banner, Sunite Left Banner, Siziwang Banner, and Abaga Banner in the global lamb isotope and local lamb isotope libraries were 68, 5, 15, and 5, and 15, 5, 15, and 5, respectively.

**Table 3:** Origin classification results of applying the 5 models to the testing set lambs according to (a) the global lamb isotopes libraries, (b) the local lamb isotopes libraries screened by AKS, and (c) the local lamb isotopes libraries screened by the approach of PCA–FD based on DD–SIMCA.

(**a**)	**Binary Discrimination Classes**				
	**LDA**	**RF**	**SVM**	**BPNN**	**KNN**
Confusion matrix (No. of testing set samples)					
True positive	17	17	17	18	17
(tpi)					
False negative	1	1	1	0	1
(fni)					
True negative	4	4	4	5	4
(tni)					
False positive	1	1	1	0	1
(fpi)					
Performance evaluation					
Sensitivity	0.9444	0.9444	0.9444	1.0000	0.9444
Specificity	0.8000	0.8000	0.8000	1.0000	0.8000
Kappa	0.7444	0.7444	0.7444	1.0000	0.7444
Accuracy	0.9130	0.9130	0.9130	1.0000	0.9130
(**b**)	**Binary Discrimination Classes**				
	**LDA**	**RF**	**SVM**	**BPNN**	**KNN**
Confusion matrix (No. of testing set samples)					
True positive	17	17	17	18	17
(tpi)					
False negative	1	1	1	0	1
(fni)					
True negative	4	5	4	5	4
(tni)					
False positive	1	0	1	0	1
(fpi)					
Performance evaluation					
Sensitivity	0.9444	0.9444	0.9444	1.0000	0.9444
Specificity	0.8000	1.0000	0.8000	1.0000	0.8000
Kappa	0.7444	0.8808	0.7444	1.0000	0.7444
Accuracy	0.9130	0.9565	0.9130	1.0000	0.9130
(**c**)	**Binary Discrimination Classes**				
	**LDA**	**RF**	**SVM**	**BPNN**	**KNN**
Confusion matrix (No. of testing set samples)					
True positive	17	17	17	18	17
(tpi)					
False negative	1	1	1	0	1
(fni)					
True negative	4	4	4	5	5
(tni)					
False positive	1	1	1	0	0
(fpi)					
Performance evaluation					
Sensitivity	0.9444	0.9444	0.9444	1.0000	0.9444
Specificity	0.8000	0.8000	0.8000	1.0000	1.0000
Kappa	0.7444	0.7444	0.7444	1.0000	0.8808
Accuracy	0.9130	0.9130	0.9130	1.0000	0.9565

## Data Availability

Data are contained within the article or supplementary material.

## Supplementary Materials

The following are available online at https://www.mdpi.com/article/10.3390/foods11060846/s1, Figure S1: Schematic diagram of the screening process about the approach of PCA–FD based on DD–SIMCA, Figure S2: 3D–score plot of (a) global lamb isotope library and (b) local lamb isotope library according to geographical origin, Figure S3: Line chart of the relationship between the sample number of the training subset and log[Det(***M****N*)] value, Figure S4: Bi-plots of (a) global lamb isotope library and (b) local lamb isotope library according to geographical origin, Table S1: Region information of lamb samples, Table S2: δ^13^C, δ^15^N, δ^2^H and δ^18^O values of all lambs (*n* = 116) from four regions.
