# Supplementary material for: Proposing Two Local Modeling Approaches for Discriminating PGI Sunite Lamb from Other Origins Using Stable Isotopes and Machine Learning
Source: Foods. 2022 Mar 16;11(6):846. doi: 10.3390/foods11060846 (PMC8954832; doi:10.3390/foods11060846)
Supplement: Supplementary file 1 [file foods-11-00846-s001.zip › foods-1616200-supplementary.pdf]

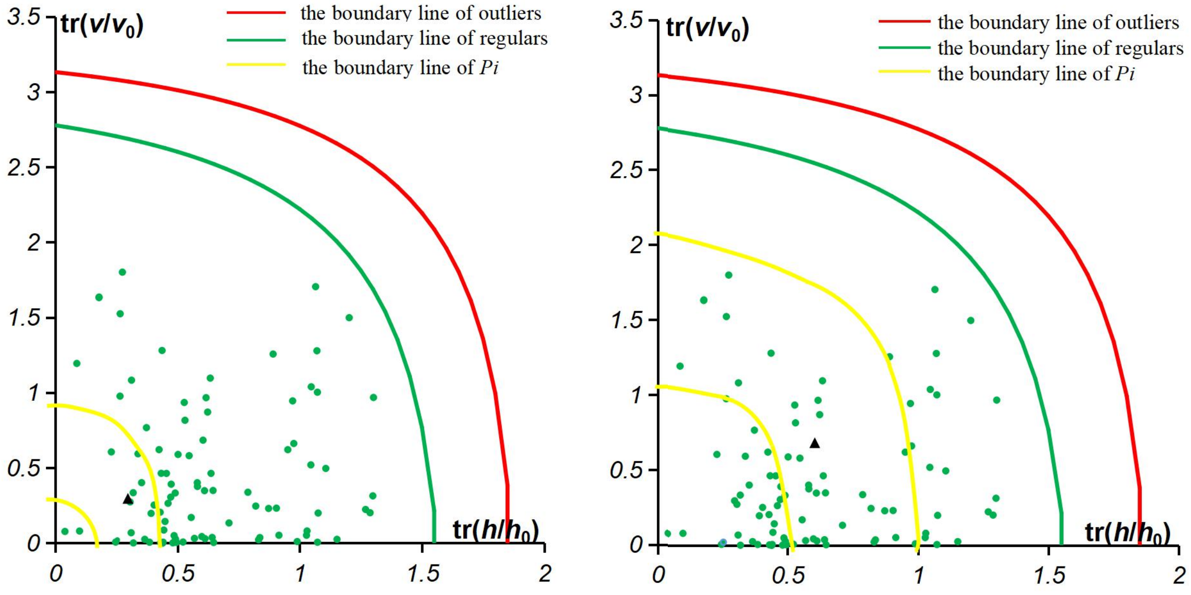

a b

Figure S1. Schematic diagram of screening process about the approach of PCA-FD based on DD-SIMCA.

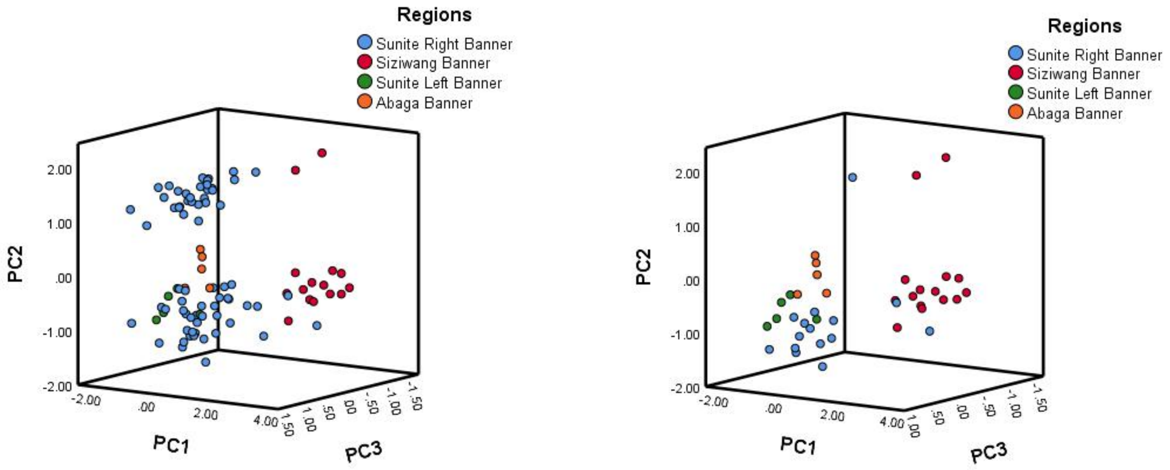

a b

Figure S2. 3D-score plot of (a) global lamb isotopes library and (b) local lamb isotopes library according to geographical origin.

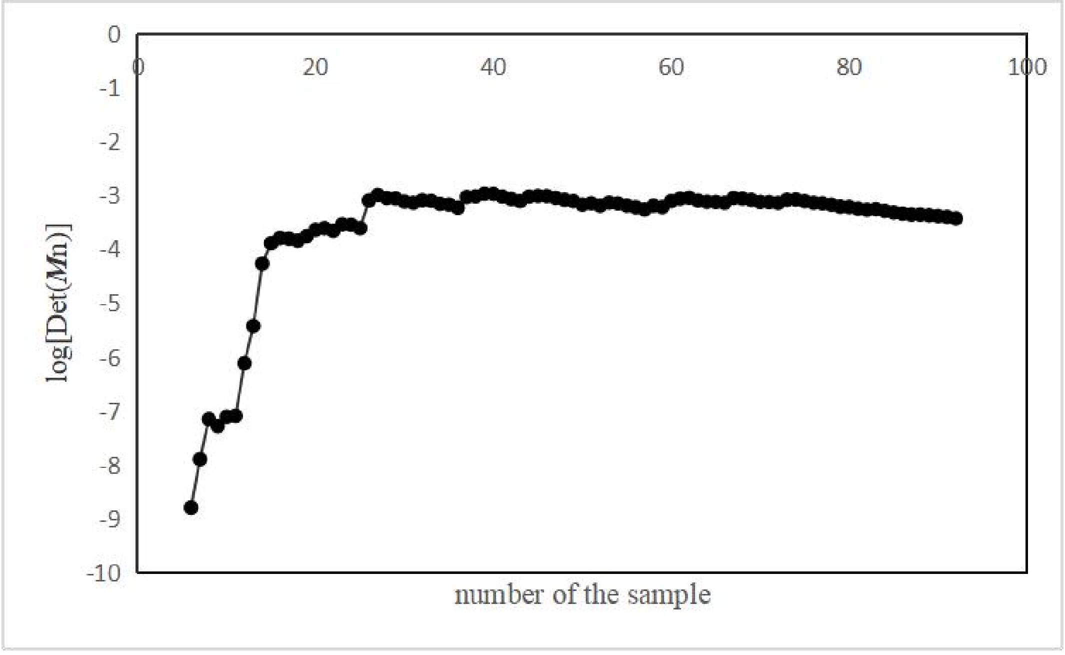

Figure S3. Line chart of the relationship between the sample number of training set subset and log[Det(M_N)] value.

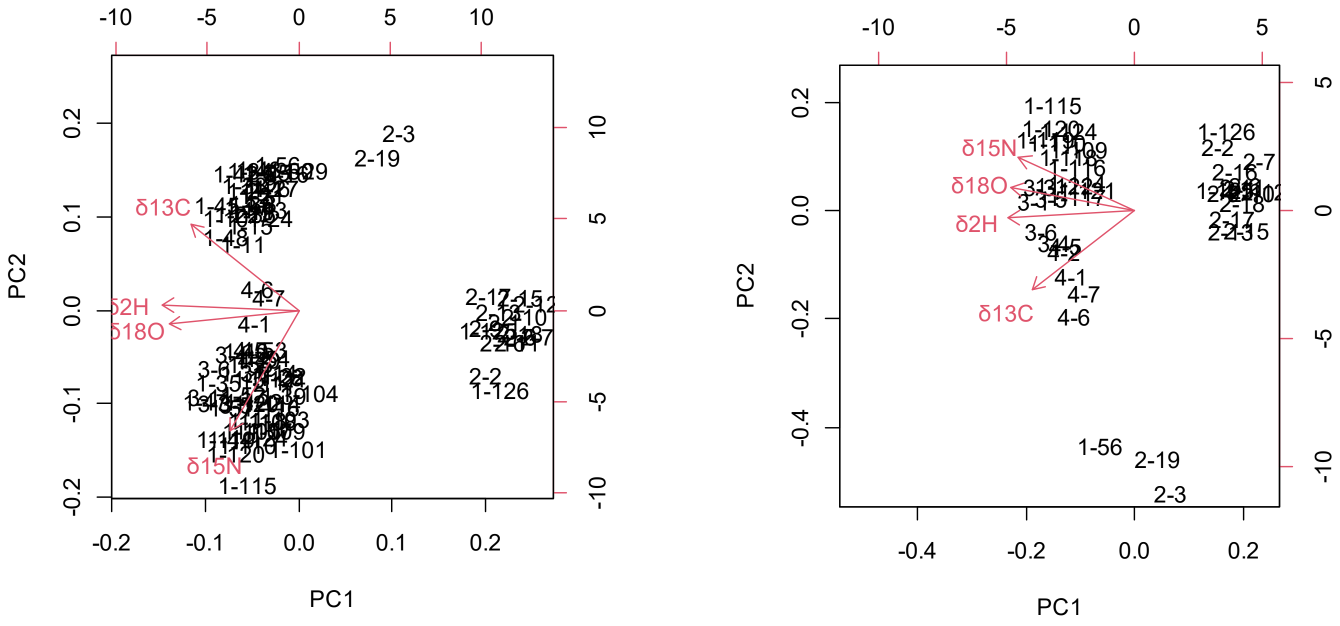

a

b

Figure S4. Bi-plots of (a) global lamb isotopes library and (b) local lamb isotopes library according to geographical origin.

Note: 1-Sunite Right Banner, 2-Siziwang Banner, 3-Sunite Left Banner, 4-Abaga Banner.

Table S1. The region information of lamb samples.

Groups	Origin	Breed	East Longitude	North Latitude	Altitude (m)	Sampling dates	Number
PGI Sunite lamb	Sunite Right Banner	Sunite sheep	112.46	43.47	1012	2020.09	84
	Sunite Left Banner	Sunite sheep	113.49	44.10	962	2019.07	7
Non-PGI lamb	Siziwang Banner	Siziwang gobi sheep	111.30	43.23	1164	2020.11	19
	Abaga Banner	Ujimqin sheep	114.37	44.18	1031	2019.04	6

Table S2. The $\delta^{13}C$, $\delta^{15}N$, $\delta^{2}H$ and $\delta^{18}O$ values of all lambs (N = 116) from four regions.

Region	$\delta^{13}C$	$\delta^{15}N$	$\delta^{2}H$	$\delta^{18}O$
Sunite Right Banner	-19.57 ± 1.59^{A}	7.38 ± 0.95^{B}	-99.88 ± 5.99^{AB}	13.52 ± 2.04^{Ab}
Sunite Left Banner	-19.44 ± 0.25^{A}	8.13 ± 0.20^{A}	-97.12 ± 1.24^{A}	15.10 ± 1.07^{Aa}
Siziwang Banner	-23.20 ± 2.09^{C}	6.23 ± 0.46^{C}	-126.85 ± 4.61^{C}	6.95 ± 1.13^{Bab}
Abaga Banner	-18.95 ± 0.30^{B}	7.50 ± 0.32^{B}	-101.42 ± 1.39^{B}	13.98 ± 0.75^{Aab}

Note: The value is given as mean ± SD; the small letters represent significant difference ($p < 0.05$), the capital letters represent extremely significant difference ($p < 0.01$) based on ANOVA analysis.
